# Supplementary material for: The interactome of intact mitochondria by cross-linking mass spectrometry provides evidence for coexisting respiratory supercomplexes
Source: Mol Cell Proteomics. 2017 Dec 8;17(2):216–32. doi: 10.1074/mcp.RA117.000470 (PMC5795388; doi:10.1074/mcp.RA117.000470)
Supplement: Supplemental Data [file supp_RA117.000470_133922_0_supp_23978_fzffwf.zip › spectra_annotation/mito_spectra_annotation/106-1-6-1-11-1.pdf]

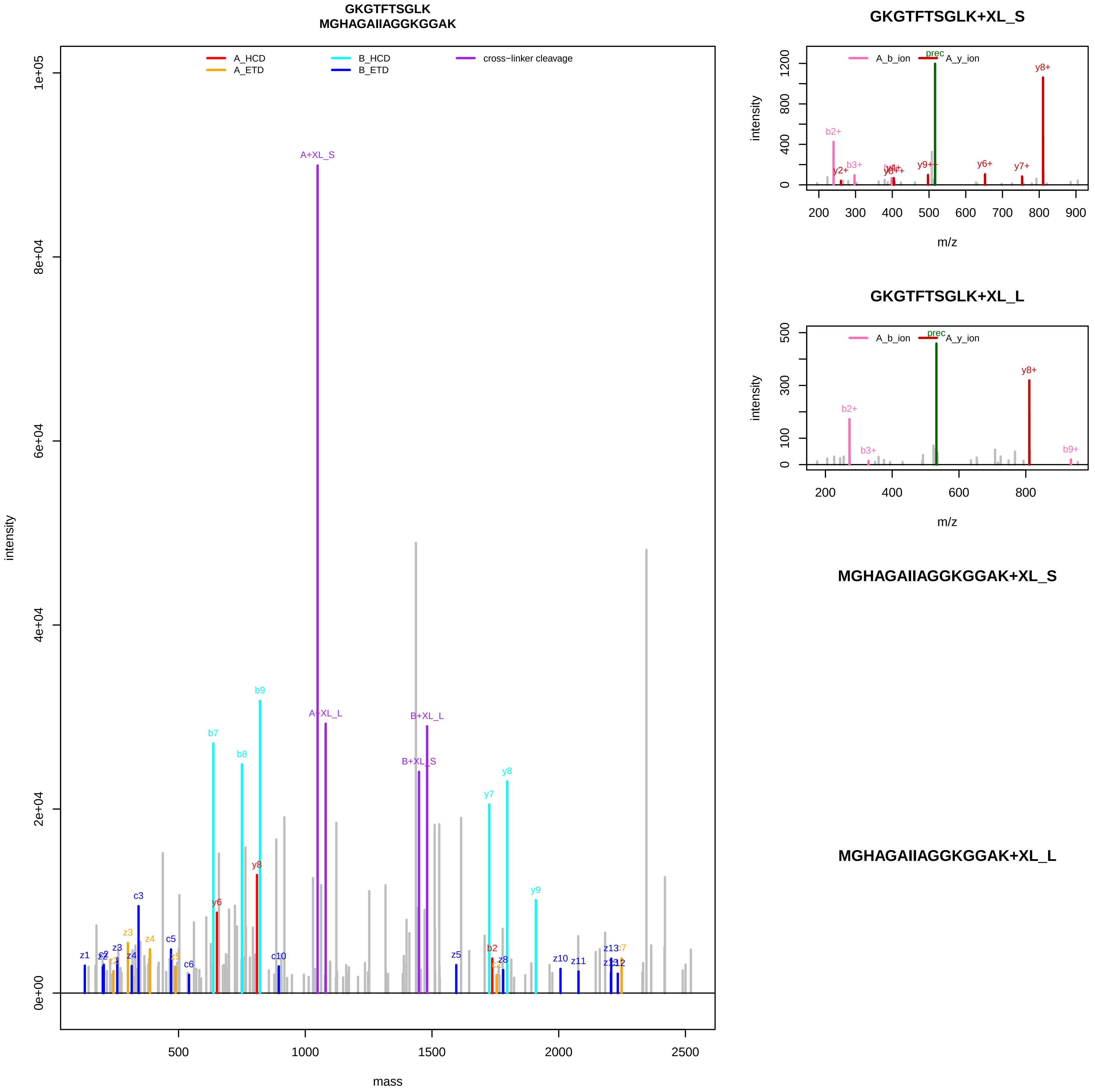
GKGTFTSGLK
MGHAGAIIAGGKGGAK
A_HCD
A_ETD
B_HCD
B_ETD
cross−linker cleavage
intensity
mass
A+XL_S
A+XL_L
B+XL_L
B+XL_S
b7
b8
b9
y6
y8
y7
y8
y9
c3
c5
c6
c10
z1
z2
z3
z4
z5
z8
z10
z11
z13
b2
GKGTFTSGLK+XL_S
A_b_ion
A_y_ion
prec
b2+
b3+
y2+
y8+
y9++
y6+
y7+
m/z
GKGTFTSGLK+XL_L
b9+
MGHAGAIIAGGKGGAK+XL_S
MGHAGAIIAGGKGGAK+XL_L
